# Distinct healthcare utilization profiles of high healthcare use tuberculosis survivors: A latent class analysis

**DOI:** 10.1371/journal.pone.0291997

**Published:** 2023-09-21

**Authors:** Kamila Romanowski, Mohammad Ehsanul Karim, Mark Gilbert, Victoria J. Cook, James C. Johnston

**Affiliations:** 1 Provincial Tuberculosis Services, British Columbia Centre for Disease Control, Vancouver, BC, Canada; 2 Department of Medicine, The University of British Columbia, Vancouver, British Columbia, Canada; 3 Faculty of Medicine, School of Population and Public Health, The University of British Columbia, Vancouver, British Columbia, Canada; 4 Centre for Health Evaluation and Outcome Sciences, St. Paul’s Hospital, Vancouver, British Columbia, Canada; 5 Clinical Prevention Services, British Columbia Centre for Disease Control, Vancouver, British Columbia, Canada; Drexel University, UNITED STATES

## Abstract

**Background:**

Recent data have demonstrated that healthcare use after treatment for respiratory tuberculosis (TB) remains elevated in the years following treatment completion. However, it remains unclear which TB survivors are high healthcare users and whether any variation exists within this population. Thus, the primary objective of this study was to identify distinct profiles of high healthcare-use TB survivors to help inform post-treatment support and care.

**Methods:**

Using linked health administrative data from British Columbia, Canada, we identified foreign-born individuals who completed treatment for incident respiratory TB between 1990 and 2019. We defined high healthcare-use TB survivors as those in the top 10% of annual emergency department visits, hospital admissions, or general practitioner visits among the study population during the five-year period immediately following TB treatment completion. We then used latent class analysis to categorize the identified high healthcare-use TB survivors into subgroups.

**Results:**

Of the 1,240 people who completed treatment for respiratory TB, 258 (20.8%) people were identified as high post- TB healthcare users. Latent class analysis results in a 2-class solution. Class 1 (n = 196; 76.0%) included older individuals (median age 71.0; IQR 59.8, 79.0) with a higher probability of pre-existing hypertension and diabetes (41.3% and 33.2%, respectively). Class 2 (n = 62; 24.0%) comprised of younger individuals (median age 31.0; IQR 27.0, 41.0) with a high probability (61.3%) of immigrating to Canada within five years of their TB diagnosis and a low probability (11.3%) of moderate to high continuity of primary care.

**Discussion:**

Our findings suggest that foreign-born high healthcare-use TB survivors in a high-resource setting may be categorized into distinct profiles to help guide the development of person-centred care strategies targeting the long-term health impacts TB survivors face.

## Introduction

People who have survived tuberculosis (TB) disease experience increased rates of all-cause mortality, respiratory disease, and cardiovascular disease post-TB [1–3]. Recent findings also demonstrate that healthcare use after treatment for respiratory TB remains elevated in the years following treatment completion [4]. This increase in post-TB healthcare utilization may be attributed to the complex healthcare needs of TB survivors, their comorbid conditions, or the consequences of TB disease [4,5]. However, there is limited data on which TB survivors are high healthcare users and whether there is any variation in comorbid conditions and healthcare utilization behaviours within this population.

A small proportion of people often account for a disproportionate amount of healthcare utilization and spending in high-resource settings [6,7]. Multimorbidity, mental health conditions, increasing age, and various indicators of low socioeconomic status have all been associated with increased healthcare use [8]. However, studies have found that high healthcare users are a heterogeneous population, with diverse medical histories, socioeconomic backgrounds, and healthcare requirements, which may contribute to the fact that only some interventions are reliably successful among these populations [9,10]. Understanding the diverse factors and unique profiles within this group is essential for tailoring effective healthcare strategies and optimizing resource allocation to address the complex needs of high healthcare users.

With the finite resources TB programs face, the potential heterogeneity in the needs of TB survivors, and the impracticality of designing interventions tailored for every individual, latent class analysis may be an avenue for TB programs to develop more targeted post-TB care models. Broadly, latent class analysis involves categorizing individuals from a heterogeneous patient population into homogenous subgroups with relatively similar characteristics or healthcare requirements, which in turn may facilitate the design of care strategies to meet the distinctive needs of each subgroup [11]. For this study, our primary objective was to identify distinct profiles of foreign-born high healthcare-use TB survivors in British Columbia, Canada. Our secondary objectives were to assess each subgroup’s most frequent reasons for post-TB healthcare use and all-cause mortality.

## Methods

### Data source and study setting

This study is part of a larger project describing TB risk among foreign-born individuals immigrating to British Columbia using deidentified linked administrative data accessed through Population Data BC [12]. Data elements include demographics, immigration information, Medical Services Plan registration and physician billings, hospital discharge, provincial disease registries, and the Provincial TB Registry [13–18].

British Columbia is a Canadian province with a low annual TB incidence of 6.0 per 100,000 residents [19]. In 2020, approximately 86% of people diagnosed with TB in British Columbia were born outside of Canada, despite representing only 22% of the population [19].

The British Columbia Medical Services Plan is the universal health insurance programme administered by the British Columbia provincial government. Enrolment with the Medical Service Plan is mandatory for all eligible residents, including those with Canadian citizenship and permanent residents who meet certain conditions [20].

The British Columbia Centre for Disease Control runs a centralized provincial TB program responsible for treating all people diagnosed with TB in the province [19]. They maintain a provincial TB registry which includes TB diagnosis and treatment data. Mandatory reporting by public health agencies, routine reporting from the centralized provincial mycobacteriology laboratory, and access to publicly funded TB medications through the provincial pharmacy make this registry virtually complete for the province’s TB disease diagnosis and treatment information.

### Study population

For the present study, we developed a retrospective cohort of people who established residency in British Columbia between January 1^st^, 1990, and December 31^st^, 2019 and completed treatment for incident respiratory TB between those dates, as coded in the Provincial TB Registry. In the absence of a consensual definition of a high healthcare user, we based our definition on percentile cut-offs as done in prior studies [21–24]. We defined high healthcare-use TB survivors as those in the top 10% of annual emergency department visits, hospital admissions, or general practitioner visits among the study population during the five-year period immediately following TB treatment completion (i.e., if a TB survivor was identified in the top 10% of users in any one of the five years following treatment, they would be classified as a high healthcare-use TB survivor).

### Latent class indicator variables

We used Andersen’s Behavioural Model of Healthcare Utilization to identify relevant latent class indicator variables to determine distinct profiles of high healthcare use in TB survivors [25]. This conceptual model proposes three sets of characteristics that influence an individual’s access and use of health services: (1) predisposing factors, which include sociocultural characteristics that exist prior to illness, such as age, sex, and socioeconomic status, (2) enabling characteristics, which include the logical aspects of obtaining care, such as the means and know how to access health service and a regular source of care, and (3) healthcare need factors, which include functional and health problems that generate the need for healthcare services [25]. For this study, all latent class indicators were defined using a two-year assessment window, which ended on the date TB treatment was completed and began two years prior (S1 Fig). This timeframe was chosen to ensure a thorough evaluation of pre-existing comorbidities and continuity of care [26,27].

For *characteristics that predispose an individual to health system access and use*, we included age at TB diagnosis, sex, immigration class, neighbourhood income decile, and TB incidence in country of birth, as proxies for sociocultural differences. All demographic and immigration information was obtained from the Immigration, Refugees, and Citizenship Canada database [17]. Neighbourhood income decile was used as a proxy for socioeconomic status and obtained from Census data. For each individual in the study population, the Census decile data closest to the year TB treatment was completed was used [28].

For *enabling characteristic that influences the likelihood of further healthcare utilization*, *we included* time since the arrival to Canada and the Continuity of Care Index (COCI). The time from arrival to Canada to TB treatment completion was used as a surrogate for healthcare literacy [29] and obtained from MSP registration data [16].

We used the COCI as a measure of a regular source of primary care. The COCI is calculated as the number of visits to the most frequent primary care physician divided by the total number of primary care visits the patient had overall. We defined a poor continuity of primary care as a Continuity of Care Index score < 0.5, and a moderate to high continuity of primary care as an Index score ≥ 0.5 [27]. Due to the limited number of people with an Index Score ≥ 0.5, we collapsed moderate (0.5–0.74) or high (0.75–1.0) continuity of care into one category [27].

Since COCI is typically calculated for people with three or more primary care visits over two years [27], we only included people with three primary care visits during the latent class assessment period in the COCI calculation. Individuals with less than three primary care visits were excluded from the COCI calculation, and data used to calculate COCI was obtained from MSP billing data [16].

For *characteristics related to future healthcare needs*, we determined the presence of six chronic comorbidities, including hypertension, diabetes, depression, chronic obstructive pulmonary disease (COPD), chronic kidney disease (CKD), and any malignancy, during the two-year covariate assessment window using validated algorithms [26]. These comorbid conditions were chosen as they are associated with increased TB risk and healthcare utilization [30,31]. The data sources and algorithms used to determine the presence of these conditions are presented in S1 Table. Lastly, we also included characteristics of TB disease as characteristics related to future healthcare needs. These variables include treatment duration and the presence of smear-positive disease, both obtained from the Provincial TB Registry.

### Statistical analysis

For our primary objective, we used latent class analysis to identify groups of high healthcare-use TB survivors. Latent class analysis is a finite mixture modelling method where observed variables, or latent class indicators, categorize individuals from a heterogeneous sample into otherwise unobserved homogenous groups [11]. Using the indicator variables described above, we fit latent class models successively using multiple random starts to replicate the maximum likelihood at least 20 times [32], starting with a one-class model and then adding additional classes iteratively. We examined model fit based on our theoretical understanding of healthcare-seeking behaviours [25], interpretability, and the following statistical information criteria: Akaike information criterion, Bayesian information criterion, consistent Akaike information criterion, and sample size adjusted Bayesian information criterion, where lower values of these indices from each successive model indicate a better fit [33]. In line with prior research, we considered Bayesian information criterion as the most reliable indicator of model fit [34,35].

Although not used to select our final model, we also examined the entropy statistic, an indicator of accurate class differentiation and posterior probabilities, and the lowest average poster probabilities [33,34]. Once we identified the best class model, we assigned each individual to a specific group based on their highest posterior class membership probabilities. Based on the profiles of the latent class indicators, we determined a name for each group and reported the prevalence of the classifying conditions among each group. For each group, we also examined the number of hospital admissions, emergency department visits, and general practitioner visits over the five-year post-TB period. We compared the results to non-high healthcare use TB survivors.

For our secondary objectives, we first examined the point prevalence for each group’s top ten most frequent reasons for post-TB healthcare use using ICD-9 and ICD-10 codes. We then calculated five-year survival probability for each group using Kaplan-Meier curves and age-standardized mortality rates, based on the World Health Organization standard population [36].

### Sensitivity analyses

We also conducted three sensitivity analyses. First, given that our primary analysis encompasses 30 years, where healthcare utilization patterns may have changed, we restricted our analysis to individuals treated for respiratory TB between 2000 and 2019. Next, we defined high healthcare use TB survivors as those in the top 5% of emergency department visits, hospital admissions, or general practitioner visits over at least one year, during the five-year period immediately following TB treatment completion. Lastly, to assess the robustness of the results concerning different assessment windows, we used a one-year assessment window for our latent class indicator variables. All analyses were conducted in R (V.4.0.5) [37]. Latent class analysis was implemented using the poLCA package [38].

### Ethical consideration and reporting

Ethical approval of this study was provided by the University of British Columbia Clinical Research Ethics Board (Certificate #H20-02454). This study exclusively used de-identified administrative data, so the need for informed consent was waived by the University of British Columbia Clinical Research Ethics Board. All data were fully anonymized before the study team accessed them. We reported this study following the guidelines for Reporting Studies Conducted Using Observational Routinely-Collected Health Data (RECORD) statement (S1 File) [39].

## Results

### Study population

**Fig 1:**
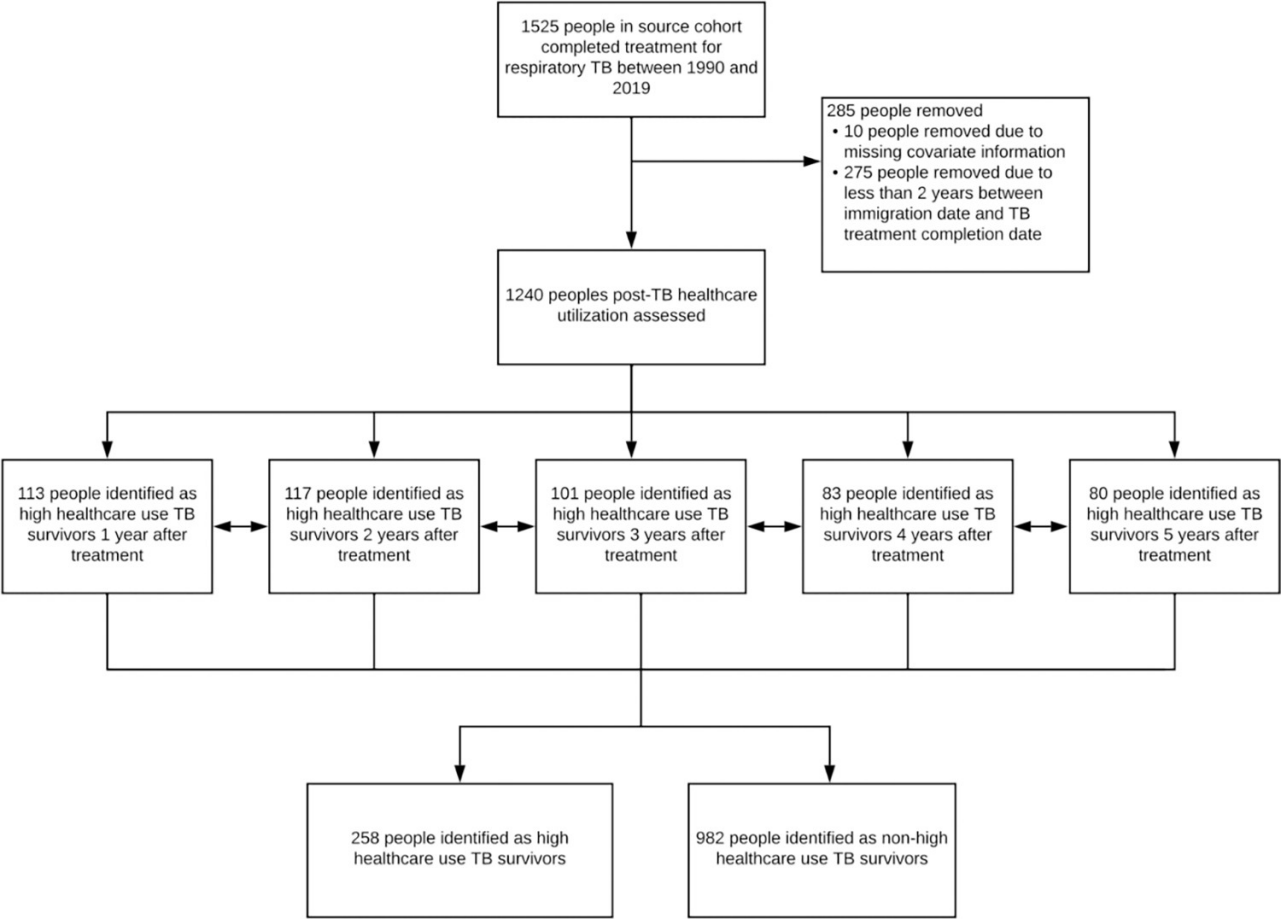
Study flowchart to identify high healthcare-use TB survivors. High healthcare-use TB survivors defined as those in the top 10% of annual emergency department visits, hospital admissions, or general practitioner visits among the study population during the five-year period immediately following TB treatment completion. Note, an individual could meet the criteria for high healthcare use across multiple years following TB treatment.

Of the 1,525 people who completed treatment for incident respiratory TB between 1990 and 2019, 1,240 were included in the study (Fig 1). In total, 258 (20.8%) people were identified as high healthcare users. The proportion of the high-users which met the various components of the high-use definition, based on the first year they met the definition, are shown in S2 Fig. The baseline characteristics of high and non-high healthcare use TB survivors are presented in Table 1. High healthcare use TB survivors had a median age of 65.5 years (IQR 46.0, 76.0), and the majority, 55.4%, were male. In contrast, non-high healthcare use TB survivors were younger, with a median age of 49 years (IQR 34.0, 69.0). The most prevalent pre-existing comorbid conditions among high-healthcare use and non-high healthcare use TB survivors included hypertension (31.8% vs 15.4%, respectively) and diabetes (25.2% vs 17.2%, respectively). Approximately 33% of high healthcare use TB survivors had immigrated to Canada within five years of their TB diagnosis, and 38.4% were in the lowest socioeconomic decile. Comparatively, 42.1% of non-high healthcare use TB survivors had immigrated to Canada within five years of their diagnosis, and 32.7% were in the lowest socioeconomic decile. Only 22.5% of high healthcare use TB survivors had moderate to high continuity of primary care, while 41.5% of non-high healthcare use TB had moderate to high continuity.

**Table 1:** Baseline characteristics for each group of high healthcare-use and non-high healthcare use TB survivors.

	All TB survivors N (%)	Non-high healthcare user TB survivors N (%)	High healthcare use TB survivors		
			All high healthcare users N (%)	Class 1: Older with pre-existing comorbidities N (%)	Class 2: Recent migrants with low continuity of care N (%)
No., %	1240	982	258	196	62
**Age at TB treatment end**, median (IQR), year	52.0 (35.0, 71.0)	49.0 (34.0, 69.0)	65.5 (46.0, 76.0)	71.0 (59.8, 79.0)	31.0 (27.0, 41.0)
**Sex**, male	717 (57.8)	574 (58.5)	143 (55.4)	133 (67.0)	10 (16.1)
**WHO tuberculosis incidence in country of origin**					
<30	33 (2.7)	26 (2.6)	7 (2.7)	<5	<5
30–100	412 (33.2)	333 (33.9)	79 (30.6)	64 (32.7)	15 (24.2)
101–200	404 (32.6)	285 (29.0)	119 (46.1)	94 (48.0)	25 (40.3)
> 200	391 (31.5)	338 (34.4)	53 (20.5)	35 (17.9)	18 (29.0)
**Immigration class**					
Family	649 (52.3)	480 (48.9)	169 (65.5)	137 (69.9)	32 (51.6)
Economic	421 (34.0)	366 (37.3)	55 (21.3)	35 (17.9)	20 (32.3)
Refugee	118 (9.5)	97 (9.9)	21 (8.1)	12 (6.1)	9 (14.5)
Other	52 (4.2)	39 (4.0)	13 (5.0)	12 (6.1)	<5
**Neighbourhood income quintile**					
1 (lowest)	420 (33.9)	321 (32.7)	99 (38.4)	74 (37.8)	25 (40.3)
2–4	553 (44.6)	450 (45.8)	103 (39.9)	74 (37.8)	29 (46.8)
5 (highest)	267 (21.5)	211 (21.5)	56 (21.7)	48 (24.5)	8 (12.9)
**≤ 5 years since arrival to Canada to TB diagnosis,**	499 (40.2)	413 (42.1)	86 (33.3)	48 (24.5)	38 (61.3)
**Pre-existing comorbidities**					
Hypertension	233 (18.8)	151 (15.4)	82 (31.8)	81 (41.3)	<5
Diabetes	234 (18.9)	169 (17.2)	65 (25.2)	65 (33.2)	<5
Depression	102 (8.2)	68 (6.9)	34 (13.2)	20 (10.2)	14 (22.6)
COPD	153 (12.3)	114 (11.6)	39 (15.1)	36 (18.4)	<5
CKD	70 (5.6)	49 (5.0)	21 (8.1)	20 (10.2)	<5
Cancer	127 (10.2)	97 (9.9)	30 (11.6)	28 (14.3)	<5
**Continuity of care index**					
Moderate to high continuity of primary care*	466 (40.1)	408 (41.5)	58 (22.5)	51 (26.0)	7 (11.3)
**Smear positive disease**	579 (46.7)	463 (47.1)	116 (45.0)	92 (46.9)	24 (38.7)
**>1 year of TB treatment**	162 (13.1)	129 (13.1)	33 (12.8)	20 (10.2)	13 (21.0)
**Follow-up**, median (IQR), years	5.0 (3.0, 5.0)	5.0 (2.6, 5.0)	5.0 (5.0, 5.0)	5.0 (4.5, 5.0)	5.0 (5.0, 5.0)

*The denominator for continuity of care is based on the total number of people who had more than 3 primary care visits over the latent class assessment window. Acronyms: Chronic obstructive pulmonary disease (COPD); chronic kidney disease (CKD). Note: Cells <5 have been supressed as per PopData protocol.

### Latent class analysis

Successive models ranging from one to six classes based on the 15 indicators of healthcare utilization were fit. A two-class solution was chosen as this was the point of plateauing of information criteria statistics. Further increases in model complexity did not yield the same decreases in Bayesian information criterion (S3 Fig) [32,33]. Moreover, the two-class solution had a clear representation of the underlying trends in the data while maintaining a manageable level of complexity for practical and insightful interpretation. For the 2-class solution, the class sample size was >10% for both classes, and entropy was >80% (S2 Table) [32,33].

### Latent class profiles

**Fig 2:**
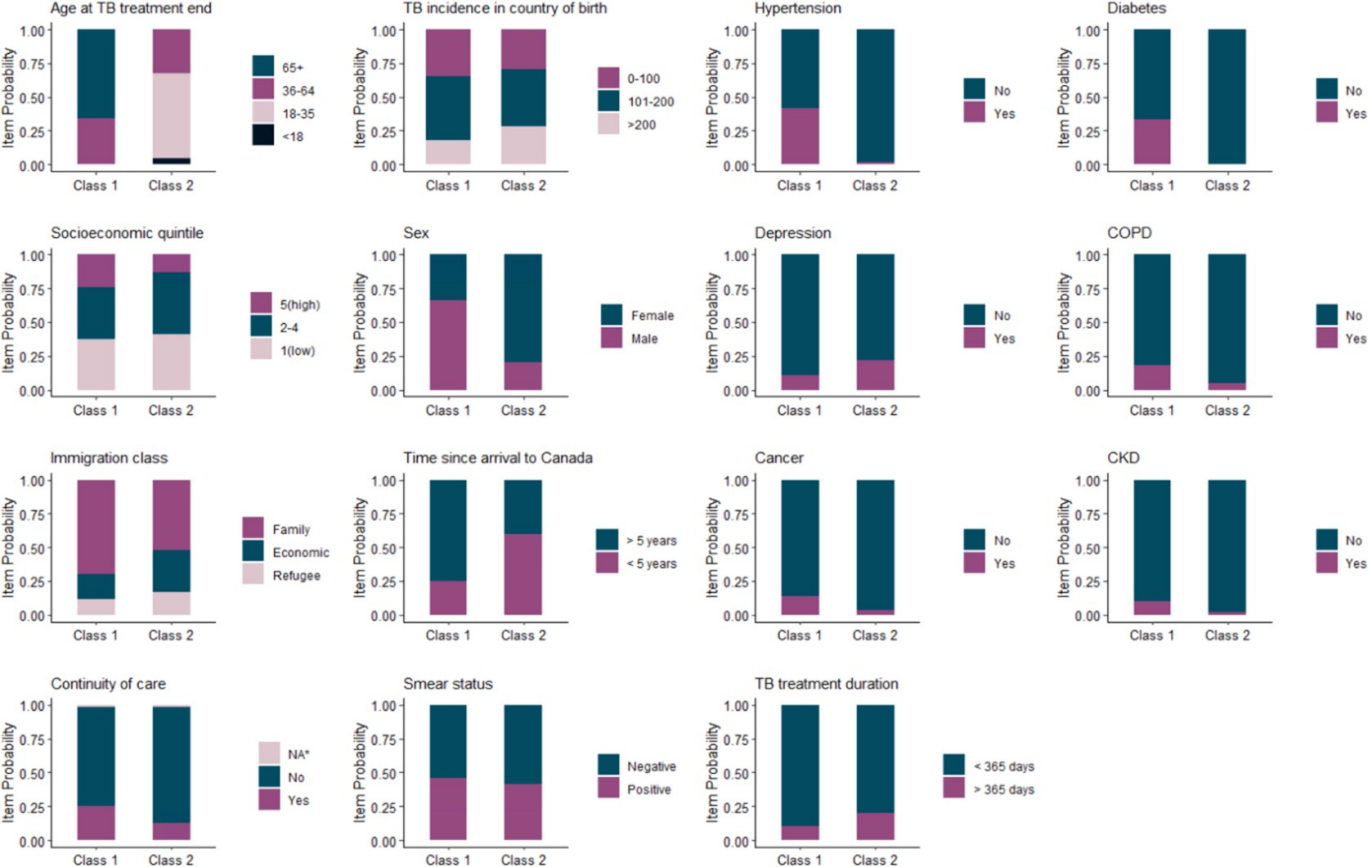
Latent profiles of high healthcare-use TB survivors. NA for continuity of primary care represents individuals who had less than 3 primary care visits over the latent class assessment window. Acronyms: chronic obstructive pulmonary disease (COPD); chronic kidney disease (CKD).

Class 1 was termed ‘older individuals with pre-existing comorbidities’ (n = 196; 76.0%) (Fig 2). This class included individuals with a median age of 71.0 (IQR 59.8, 79.0). They had a higher probability of pre-existing hypertension and diabetes (41.3% and 33.2%, respectively) and a high probability (67.0%) of being male.

Class 2 was termed ‘younger recent migrants with low continuity of primary care’ (n = 62; 24.0%). This class comprised of individuals with a median age of 31.0 (IQR 27.0, 41.0). Individuals in this group had a high probability (61.3%) of immigrating to Canada within five years of their TB diagnosis and a low probability (11.3%) of moderate to high continuity of primary care. They also had a higher probability of pre-existing depression (22.6%) and being female (83.9%).

Individuals in both Class 1 and Class 2 had similar probabilities for smear-positive disease (46.9% vs. 38.7%, respectively), while Class 2 has a higher probability of TB treatment duration over 365 days (10.2% vs. 21.0%, respectively).

### Post-TB healthcare utilization

Measures of post- TB healthcare utilization varied across groups (Table 2). Overall, individuals in Class 1 had a median of 22.7 (IQR 16.6, 30.2) general practitioner visits, 0.8 (IQR 0.4, 1.5) hospital admissions, and 0.8 (IQR 0.3, 1.8) emergency department visits per person, per year over the 5-year post-TB follow-up. Individuals in Class 2 had a median of 19.0 (IQR 14.6, 25.0) general practitioner visits, 0.4 (IQR 0.2, 0.4) hospital admissions, and 0.8 (IQR 0.2, 1.4) emergency department visits per person per year. In contrast, non-high healthcare use TB survivors had a median of 6.8 (IQR 3.6, 10.8) general practitioner visits, 0.4 (IQR 0.2, 0.8) hospital admissions, and 0.4 (IQR 0.2, 0.8) emergency department visits per person per year.

**Table 2:** 5-year post-TB healthcare utilization and all-cause mortality.

	Non-high healthcare use tuberculosis survivors	High healthcare use tuberculosis survivors	
		Class 1: Older with pre-existing comorbidities	Class 2: Recent migrants with low continuity of care
**Post-TB healthcare utilization** ^a^			
Median number of hospital admissions (IQR)	0.4 (0.2, 0.8)	0.8 (0.4, 1.5)	0.4 (0.2, 0.4)
Median number of emergency department visits (IQR)	0.4 (0.2, 0.8)	0.8 (0.3, 1.8)	0.8 (0.2, 1.4)
Median number of general practitioner visits (IQR)	6.8 (3.6, 10.8)	22.7 (16.6, 30.2)	19.0 (14.6, 25.0)
**All-cause mortality**			
Death, n (%)	53 (5.4)	31 (15.8)	<5
Age at death, median (IQR), years	81.0 (73.5, 86.0)	84.0 (70.0, 88.0)	39 (37.8, 44.5)
Age standardized mortality rate, per 1,000 standard population (95% CI)	10.0 (6.6, 13.3)	8.4 (4.9, 11.9)	48.1 (26.5, 69.7) ^b^

^a^ Per person, per year

^b^ As this rate is based on <20 events, it should be interpreted with caution.

Note: Cells <5 have been supressed as per PopData protocol.

### Top reasons for healthcare use

**Fig 3:**
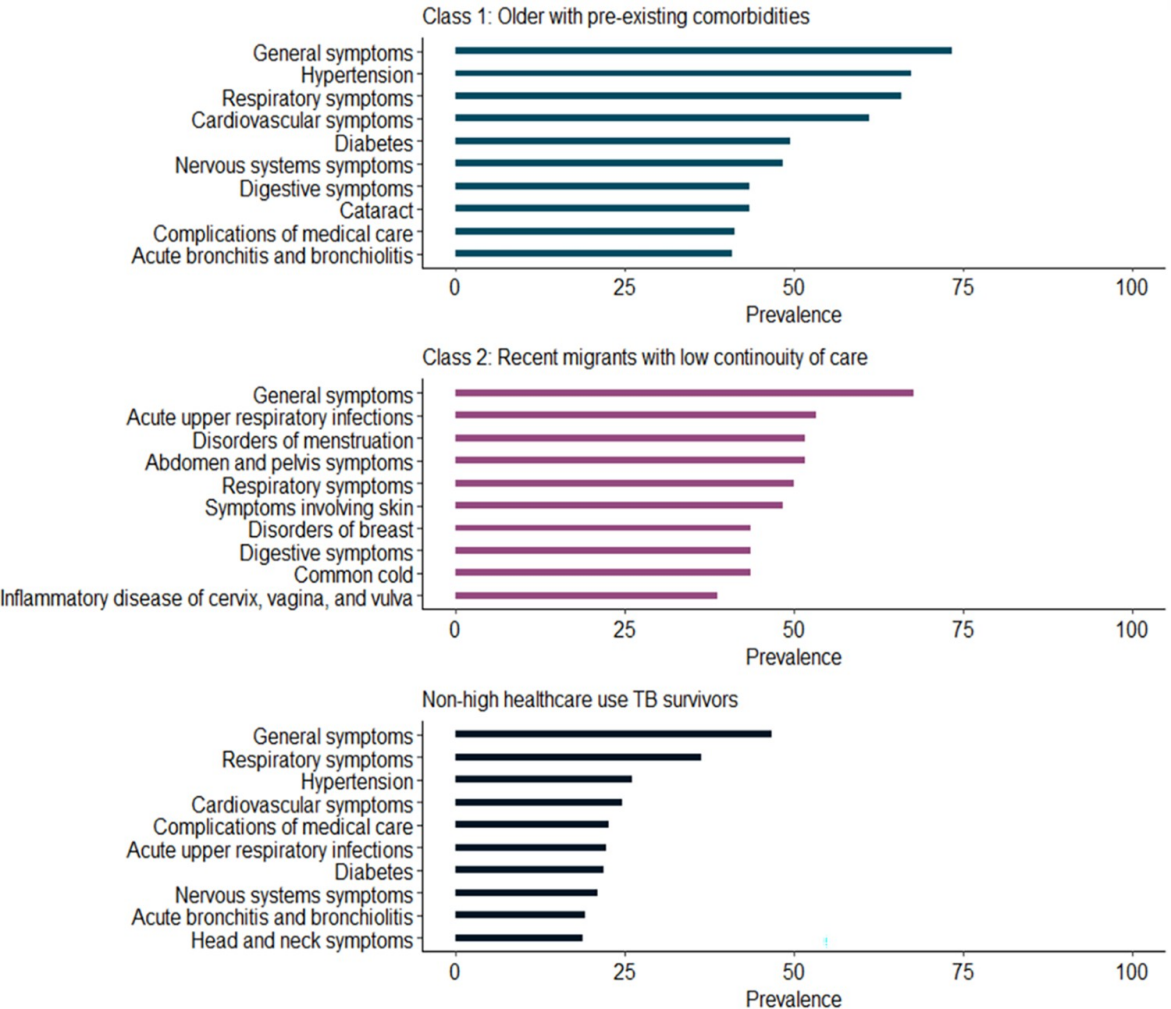
Prevalence of the top 10 reasons for healthcare visits for each group of high healthcare-use TB survivors and non-high healthcare users.

General signs and symptoms were the leading reason for healthcare visits for each group of high healthcare-use TB survivors and non-high healthcare users (Fig 3). More than 60% of individuals in Class 1 visited a healthcare provider for hypertension, respiratory symptoms, and cardiovascular symptoms. Roughly 50% also visited a healthcare provider for diabetes. Over 50% of people in Class 2 also visited a healthcare provider for respiratory reasons.

### All-cause mortality

**Fig 4:**
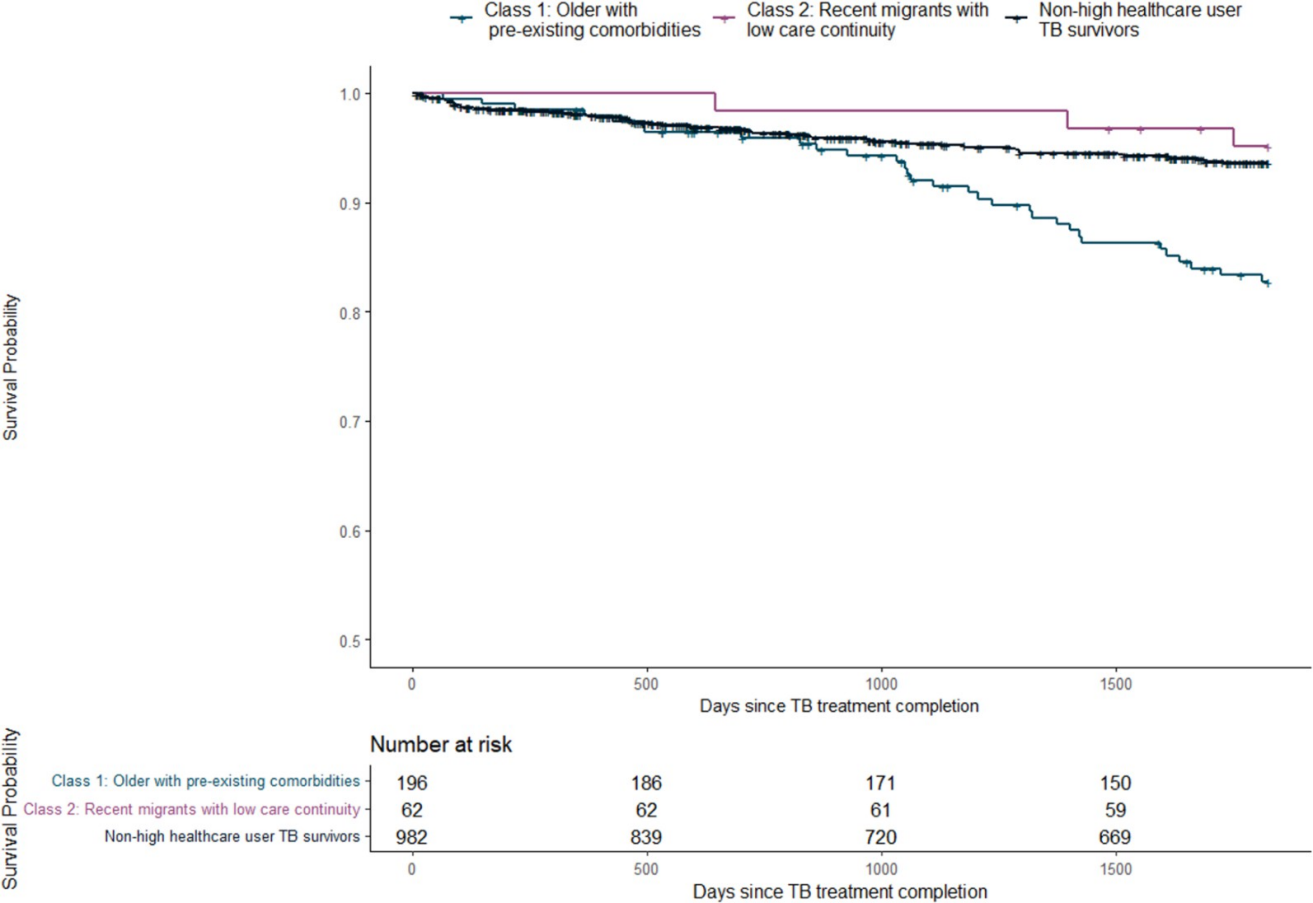
Five-year survival probability for each group of high healthcare-use TB survivors and non-high healthcare users.

In total, 31.0 (15.8%) high healthcare users in Class 1 died during the five-year follow-up, while less than five people died in Class 2 (Table 2). The median age of death for individuals in Class 1 was 84.0 years (IQR 70.0, 88.0), while the median age for those in Class 2 was 39.0 (IQR 37.8, 44.5). For non-high healthcare users, 53.0 (5.4%) individuals died during the five-year follow-up, and their median age was 81.0 (IQR 73.5, 86.0). The five-year survival probability for each group of high healthcare-use TB survivors and non-high healthcare users is presented in Fig 4.

When comparing age-standardized mortality rates, the mortality rate was 8.4 (95% CI 4.9, 11.9) per 1,000 standard population for Class 1, 48.1 (95% CI 26.5, 69.7) per 1,000 standard population for Class 2, and 10.0 (95% CI 6.6, 13.3) per 1,000 standard population for non-high healthcare users (Table 2). However, as the age-standardized mortality rate for Class 2 is based on less than 20 events, these results should be interpreted with caution.116 The top three leading causes of mortality were neoplasms, circulatory diseases, and respiratory diseases (S4 Fig).

### Sensitivity analyses

Restricting our analysis to individuals who completed treatment for respiratory TB between 2000 and 2019, modifying our definition of high healthcare use, and limiting our assessment window to one year resulted in findings consistent with our primary analysis. The latent profiles for each sensitivity analysis are presented in the Supplementary Appendix (S5–S7 Figs).

## Discussion

Using latent class analysis, we identified two distinct profiles of foreign-born high healthcare-use TB survivors: older individuals with pre-existing comorbidities and younger recent migrants with low continuity of primary care. By identifying the key variables that define each profile, our study offers data to help guide the development of person-centred care strategies targeting the long-term health impacts TB survivors face in high-resource settings.

In our cohort, high healthcare-use TB survivors with pre-existing conditions used more healthcare than the younger high healthcare-use subgroup. This is likely because the probability of being a frequent healthcare user increases with age and the number of health conditions; the higher the burden of chronic disease, the more frequent the visits to emergency departments and general practitioners [21]. As recent research has also highlighted, multimorbidity is common among TB survivors, with an estimated two-fold higher prevalence of at least one chronic health condition among people with TB [40–42]. Care coordination, an organization-focused intervention designed to match the health and social needs of people living with multimorbidity, may be a potential solution for individuals undergoing TB treatment with multimorbidity as it can lead to better communication, efficient resource utilization, and improved quality of life [43,44].

Our analysis identified that only 22.5% of high healthcare-use TB survivors had moderate to high continuity of primary care. Comparatively, a random sample of 50,000 people from our source cohort indicated 49% of people who immigrate to Canada have moderate to high continuity of primary care, suggesting that, overall, TB survivors have lower continuity of primary care. The probability of moderate or high continuity of primary care further decreased to 11.3% among people in Class 2 (younger recent migrants with low continuity of primary care). Notably, low continuity of primary care was a key identifying variable between Class 2 members and non-high healthcare use TB survivors. While this is likely, in part, a limitation of administrative data and our inability to account for specific measures of social determinants of health and lifestyle factors, it does suggest continuity of care is an important variable in identifying high- healthcare use TB survivors. Moreover, it is a variable that can be presumably identified at diagnosis quite easily.

Our findings support research showing that people who have recently immigrated to Canada are less likely to have a family physician and are more likely to use walk-in health services or emergency departments [45]. In Canada, access to primary care is challenging, with approximately 18% of British Columbians reporting they do not have access to or are not attached to a regular healthcare provider [46,47]. Challenges accessing care are further compounded for older adults with multiple comorbidities, new migrants whose primary language is not the language in which care is provided, or people whom the healthcare system has historically mistreated [48]. Language and cost barriers, insurance waiting periods, lack of knowledge on navigating the healthcare system, and a lack of culturally relevant and culturally safe care all contribute to this inequitable access [49]. Given that the overwhelming majority of high healthcare-use TB survivors did not have primary care attachment, we recommend TB programs ensure people undergoing treatment for respiratory TB, particularly those who have recently immigrated or those with comorbid conditions, are linked to stable primary care before they have completed treatment [50,51].

Our results should be interpreted considering limitations. First, while using provincial TB data and near-complete capture of hospital admissions and physician encounters was a significant strength of our study, linked administrative data does not capture necessary measures of social determinants of health and other health service access and use indicators. For example, we could not capture detailed information on socioeconomic status and did not have the necessary variables to calculate a deprivation index, thus, we had to use neighbourhood income decile as a proxy variable [52]. Additionally, we could not include information on socio-cultural or lifestyle factors that may influence healthcare use or account for disease severity. While income is a powerful indicator of health, it does not adequately capture all domains of socioeconomic status or access to care [52]. For example, studies have shown that individuals from lower-income households are more likely to make frequent visits to the emergency department [53,54]. This relationship is often attributed to barriers in accessing primary care and limited resources for managing chronic conditions, which can lead individuals to seek acute care in emergency settings [53,54].

Next, for our primary analysis, we removed 275 (18%) people as they had less than two years between immigration and TB treatment completion. However, when we conducted our sensitivity analysis using a one-year latent variable assessment period, only 107 (7%) people were excluded, and this analysis resulted in nearly identical results. Third, in Canada, approximately 30% of TB diagnoses among people who are foreign-born are temporary visa holders, including tourists, visitors, students, or temporary workers [55]. Unfortunately, our data only includes information on individuals who have received permanent landing status, thus we were unable to include these individuals in our analysis. Also, our results may be less generalizable to lower resource settings with a higher TB incidence or to settings that do not have universal health insurance programmes [56]. Finally, these results span a 30-year period where health system utilization may have changed. However, our sensitivity analysis restricting our timeline provided nearly identical results.

Our findings suggest two distinct profiles of foreign-born, high healthcare-use TB survivors in high-resource settings: older individuals with pre-existing comorbidities and younger recent migrants with low continuity of primary care. Across these groups, measures of post-TB healthcare utilization and mortality varied. These findings provide important insights that may help guide the development of person-centred care strategies targeting the long-term health impacts of TB.

## Supporting information

S1 Fig Latent class analysis study periods. (PDF)

S2 Fig Proportion of high-users which met the various components of the high-use definition, based on the first year they met the definition. (PDF)

S3 Fig Evaluating class solutions: model fit criteria. (PDF)

S4 Fig Top five causes of death for high and non-high healthcare use TB survivors. (PDF)

S5 Fig Latent profiles of high healthcare use TB survivors who completed treatment for respiratory TB between 2000 and 2019. (PDF)

S6 Fig Latent profiles of high healthcare use tuberculosis survivors, where high healthcare use is defined as those in the top 5% of emergency department visits, hospital admission, or general practitioner visits among tuberculosis survivors. (PDF)

S7 Fig Latent profiles of high healthcare use TB survivors, where the latent indicator assessment window ended on the date TB treatment was completed and began one year prior. (PDF)

S1 Table ICD9 and 10 codes used to identify comorbid conditions. (PDF)

S2 Table Evaluating class solutions: diagnostic criteria. (PDF)

S1 File RECORD checklist. (PDF)
