# Supplementary figures and images for: Distinct healthcare utilization profiles of high healthcare use tuberculosis survivors: A latent class analysis
Source: PLoS One. 2023 Sep 21;18(9):e0291997. doi: 10.1371/journal.pone.0291997 (PMC10513257; doi:10.1371/journal.pone.0291997)

**Supplemental Figure 1.** Latent class analysis study periods

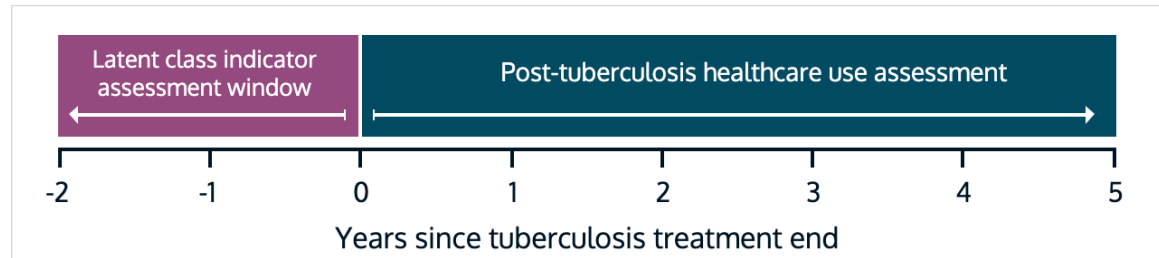

Supplement: S1 Fig — (PDF) [file pone.0291997.s001.pdf]
