# Supplementary material for: Distinct healthcare utilization profiles of high healthcare use tuberculosis survivors: A latent class analysis
Source: PLoS One. 2023 Sep 21;18(9):e0291997. doi: 10.1371/journal.pone.0291997 (PMC10513257; doi:10.1371/journal.pone.0291997)

Supplemental Figure 2. Proportion of high-users who met the various components of the high-use definition, based on the first year they met the definition.

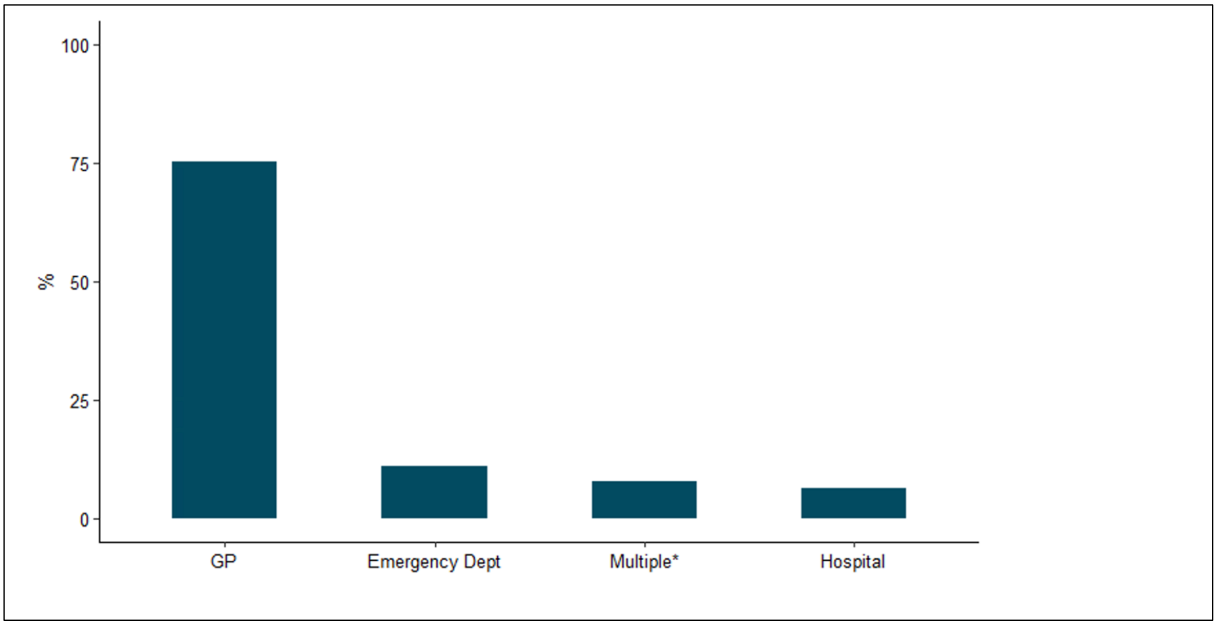

Supplement: S2 Fig — (PDF) [file pone.0291997.s002.pdf]
