# Supplementary material for: Distinct healthcare utilization profiles of high healthcare use tuberculosis survivors: A latent class analysis
Source: PLoS One. 2023 Sep 21;18(9):e0291997. doi: 10.1371/journal.pone.0291997 (PMC10513257; doi:10.1371/journal.pone.0291997)
Supplement: S3 Fig — (PDF) [file pone.0291997.s003.pdf]

Supplementary Figure 3. Evaluating class solutions: model fit criteria

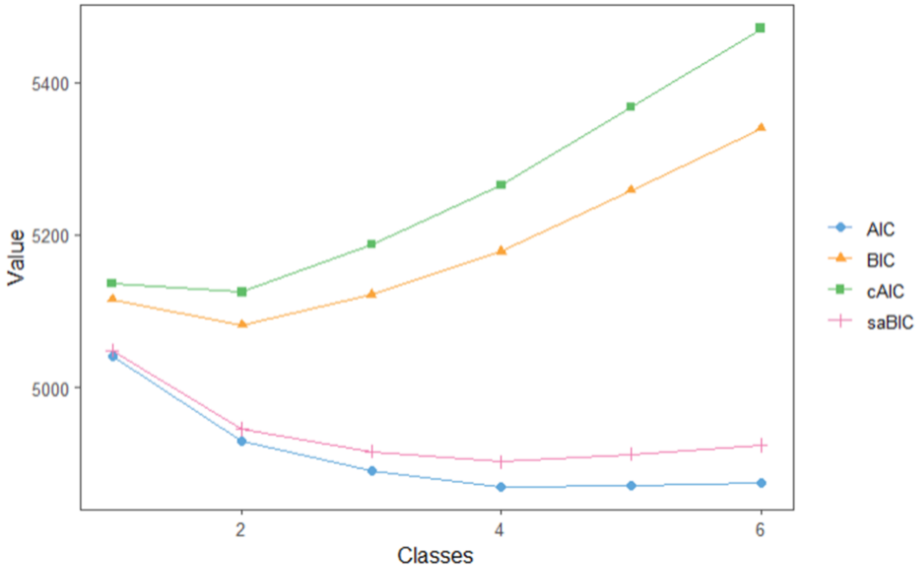

AIC = Akaike information criterion; BIC = Bayesian information criterion; cAIC = consistent Akaike information criterion; saBIC = sample-size adjusted Bayesian information criterion
