# Supplementary material for: Distinct healthcare utilization profiles of high healthcare use tuberculosis survivors: A latent class analysis
Source: PLoS One. 2023 Sep 21;18(9):e0291997. doi: 10.1371/journal.pone.0291997 (PMC10513257; doi:10.1371/journal.pone.0291997)
Supplement: S4 Fig — (PDF) [file pone.0291997.s004.pdf]

Supplementary Figure 4. Top five causes of death for high and non-high healthcare use TB survivors

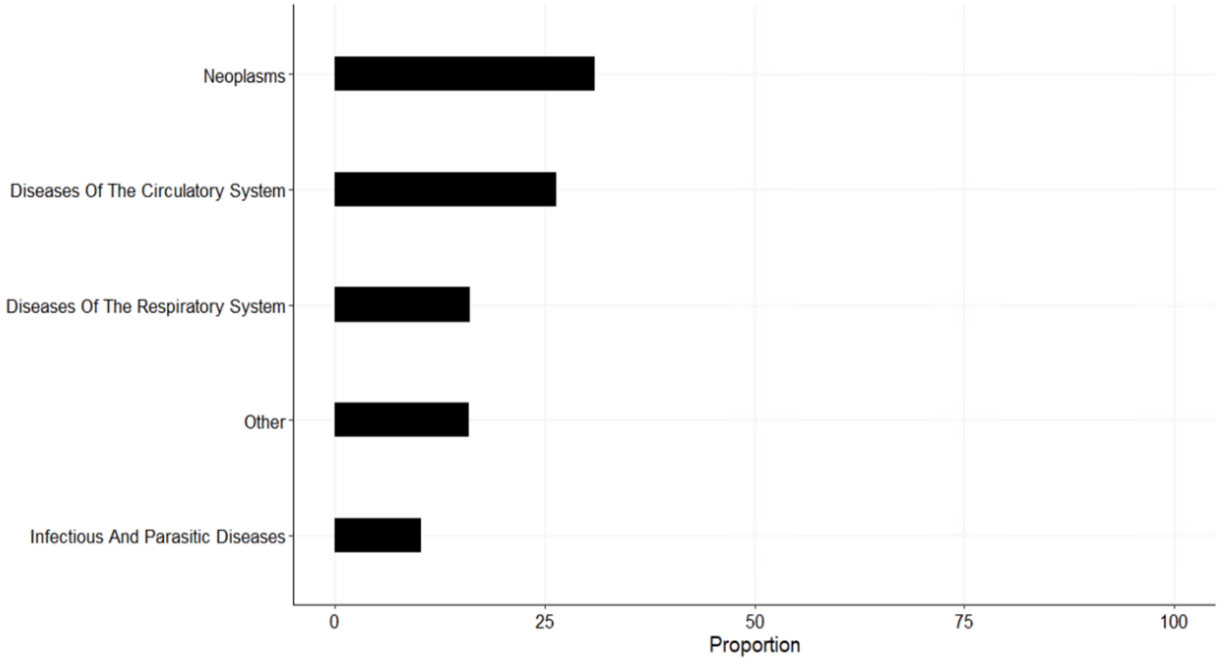

**Leading causes of death**	**N (%)**
All deaths	87
Neoplasms	27 (31.0)
Diseases of the circulatory system	23 (26.4)
Diseases of the respiratory system	14 (16.1)
Other	14 (16.1)
Infectious and parasitic diseases	9 (10.3)

* Due to small cell counts and PopData privacy agreements, we are unable to present death-cause data stratified by healthcare utilization class
