# Supplementary material for: Distinct healthcare utilization profiles of high healthcare use tuberculosis survivors: A latent class analysis
Source: PLoS One. 2023 Sep 21;18(9):e0291997. doi: 10.1371/journal.pone.0291997 (PMC10513257; doi:10.1371/journal.pone.0291997)
Supplement: S5 Fig — (PDF) [file pone.0291997.s005.pdf]

Supplementary Figure 5. Latent profiles of high healthcare use TB survivors who completed treatment for respiratory TB between 2000 and 2019

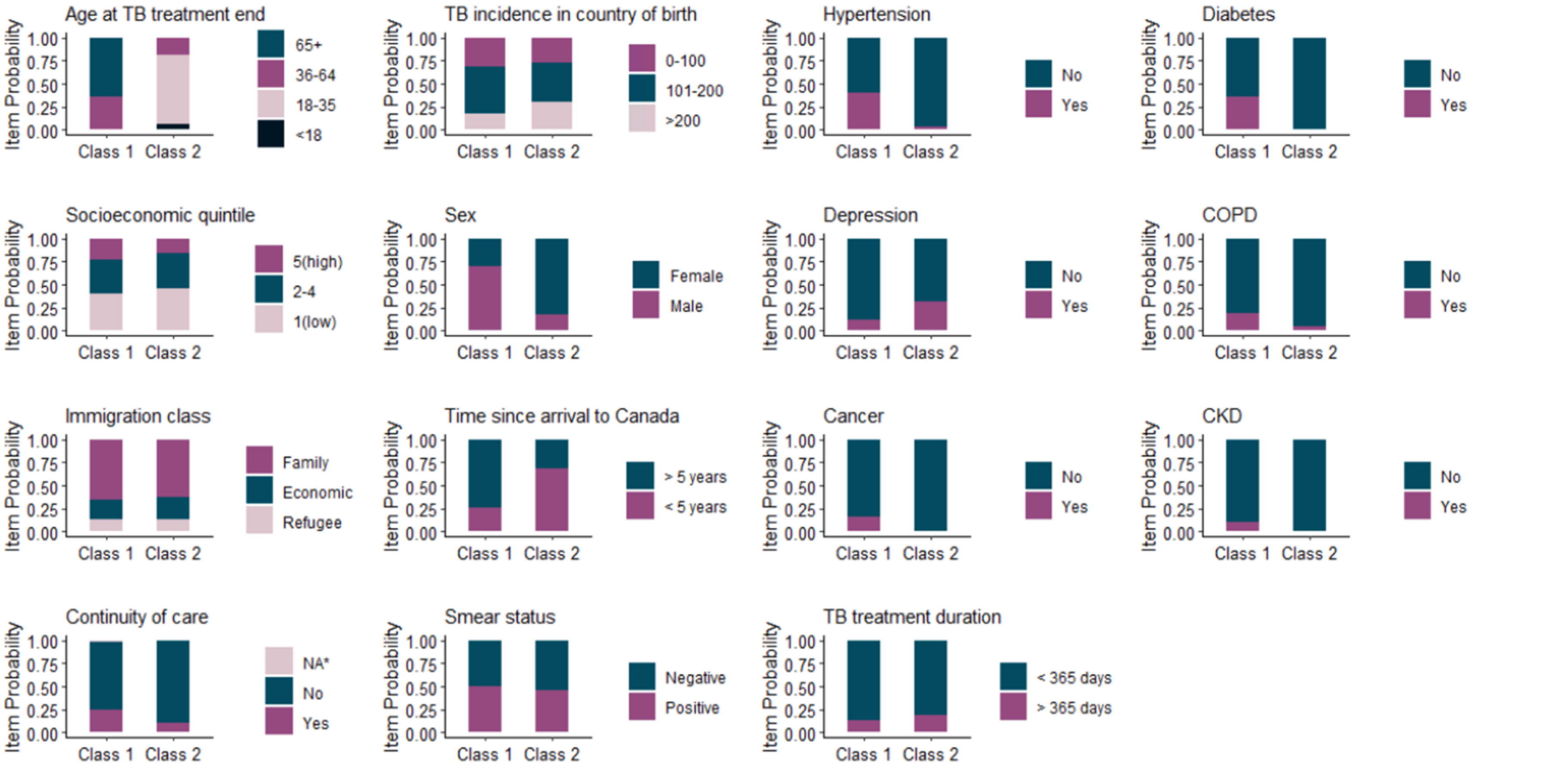

**NA for continuity of primary care represents individuals who had less than 3 primary care visits over the latent class assessment window. Acronyms: chronic obstructive pulmonary disease (COPD); chronic kidney disease (CKD)*
