# Supplementary material for: Distinct healthcare utilization profiles of high healthcare use tuberculosis survivors: A latent class analysis
Source: PLoS One. 2023 Sep 21;18(9):e0291997. doi: 10.1371/journal.pone.0291997 (PMC10513257; doi:10.1371/journal.pone.0291997)
Supplement: S7 Fig — (PDF) [file pone.0291997.s007.pdf]

Supplementary Figure 7. Latent profiles of high healthcare use TB survivors, where the latent indicator assessment window ended on the date TB treatment was completed and began one year prior.

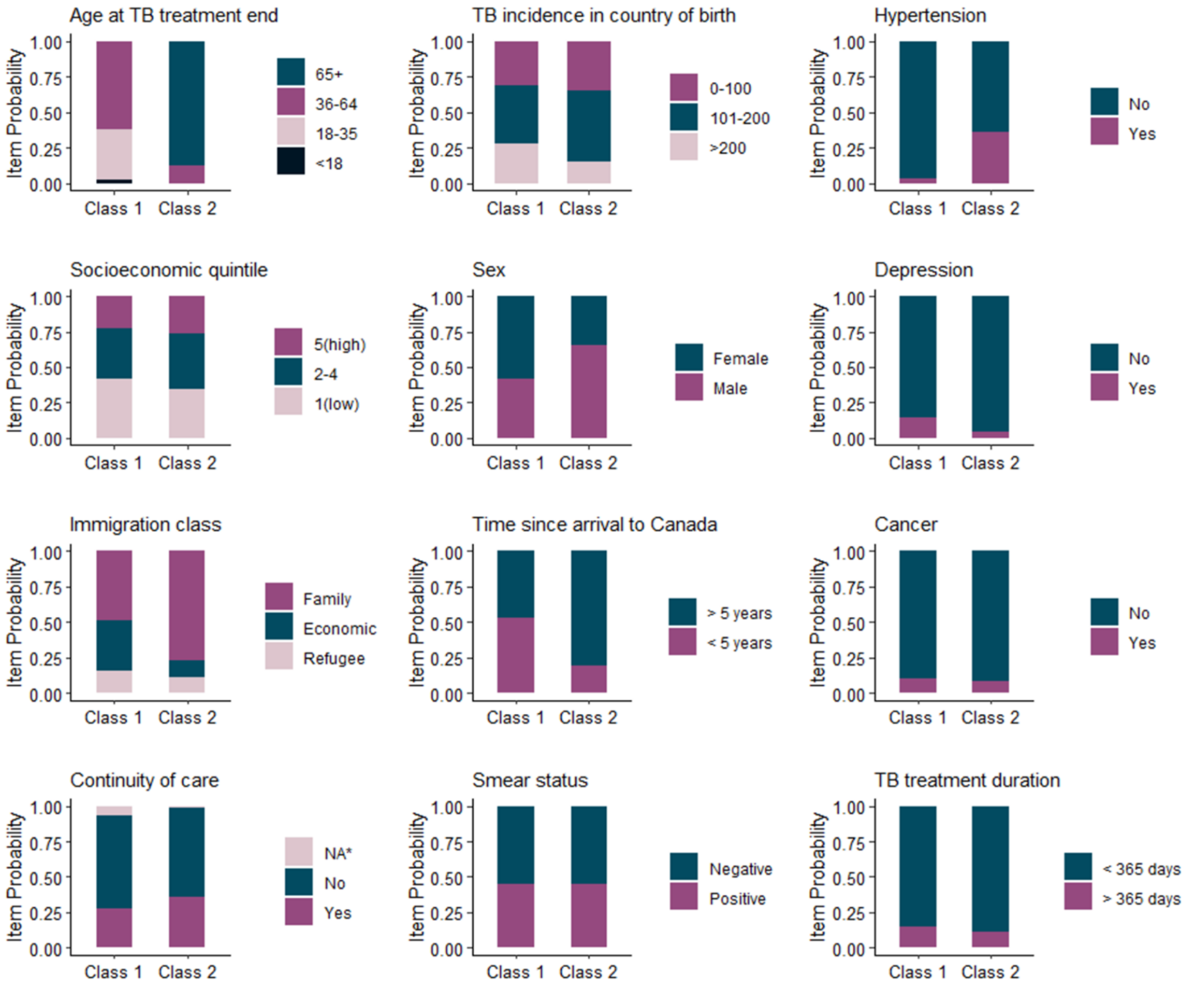

**NA for continuity of primary care represents individuals who had less than 3 primary care visits over the latent class assessment window. Acronyms: chronic obstructive pulmonary disease (COPD); chronic kidney disease (CKD)*
