# Supplementary material for: Distinct healthcare utilization profiles of high healthcare use tuberculosis survivors: A latent class analysis
Source: PLoS One. 2023 Sep 21;18(9):e0291997. doi: 10.1371/journal.pone.0291997 (PMC10513257; doi:10.1371/journal.pone.0291997)
Supplement: S1 Table — (PDF) [file pone.0291997.s008.pdf]

Supplementary Table 1. ICD9 and 10 codes used to identify comorbid conditions.

Pre-existing condition	Algorithm	ICD-9	ICD-10
Hypertension	1 hospitalization or 2 MSP claims in 2 years or less	401- 405	I10, I11, I12, I13, I15
Diabetes	1 hospitalization or 2 MSP claims in 2 years or less	250	E10 - E14
Depression	1 hospitalization or 2 MSP claims in 2 years or less	296, 300, 309, 311	F20, F31- F34, F41, F43
Chronic obstructive pulmonary disease	1 hospitalization or 3 MSP claims in 1 year	416, 490, 491, 492, 494 - 506, 508	J27, J40- J44, J46, J47, J60 - J68
Chronic kidney disease	1 hospitalization or 2 MSP claims in 2 years or less	583 – 586, 592, 593	N00- N23
Any malignancy	1 hospitalization or 2 MSP claims in 2 years or less	153, 154, 162, 163, 174, 180, 185, 196- 199, 230, 233	C18 – C21, C33, C34, C45, C50, C53, C61, C77 – C80, D01, D05-D07
