# Supplementary material for: Distinct healthcare utilization profiles of high healthcare use tuberculosis survivors: A latent class analysis
Source: PLoS One. 2023 Sep 21;18(9):e0291997. doi: 10.1371/journal.pone.0291997 (PMC10513257; doi:10.1371/journal.pone.0291997)
Supplement: S2 Table — (PDF) [file pone.0291997.s009.pdf]

Supplementary Table 2. Evaluating class solutions: diagnostic criteria

Classes	Smallest class count (n)	Smallest class size (%)	Entropy
1	258	100.0	-
2	62	24.0	0.83
3	44	17.1	0.87
4	42	16.3	0.89
5	7	2.7	0.92
6	6	2.3	0.92
